# Is There a Higher Risk of Obstructive Sleep Apnea among Individuals with Lower-Limb Compared to Those with Upper-Limb Lymphedema?

**DOI:** 10.1055/s-0046-1819596

**Published:** 2026-11-03

**Authors:** Hossein Yarmohammadi, Sam Ashtiani, Sina Shahshenas, Masood Soltanipur, Amirhossein Bahanesteh, Hamidreza Mirabi, Fereshteh Abbasvandi, Ali Montazeri, Zahra Sheikhi

**Affiliations:** 1 Integrative Oncology Research Group, Motamed Cancer Institute, TehranIran; 2 Faculty of Medicine, University of Central Lancashire, Preston, LancashireUnited Kingdom; 3 Student Research Committee, Shahed University Faculty of Medical Sciences, TehranIran; 4 Department of Quality of Life, Motamed Cancer Institute, TehranIran; 5 Student Research Committee, Shahid Beheshti University of Medical Sciences, TehranIran; 6 Department of Otorhinolaryngology, Shahid Beheshti University of Medical Sciences, TehranIran; 7 Department of Advanced Therapy Medicinal Products (ATMPs), Motamed Cancer Institute, TehranIran; 8 Breast Cancer Research Center, Motamed Cancer Institute, TehranIran; 9 Academic Center for Education, Culture, and Research (ACECR), Health Metrics Research Center, TehranIran

**Keywords:** lymphedema, obstructive sleep apnea, lower limb lymphedema, sleep quality, STOP-BANG, Epworth Sleepiness Scale, quality of life

## Abstract

**Background:**

Lymphedema, a chronic condition caused by impaired lymphatic drainage, can significantly affect patients' quality of life. While its impact on sleep quality is recognized, the association between lymphedema and obstructive sleep apnea (OSA) remains underexplored. This study investigated whether individuals with lower-limb lymphedema (LLL) are at a higher risk of OSA compared to those with upper-limb lymphedema (ULL) and healthy controls.

**Materials and Methods:**

This cross-sectional study included 91 participants: 32 with ULL, 29 with LLL, and 30 healthy controls. Lymphedema was diagnosed based on excess volume (EV) and EV percentage (EVP) using standardized circumference measurements and the cylinder formula. Sleep quality and OSA risk were assessed using validated questionnaires: the Pittsburgh Sleep Quality Index (PSQI), the 12-Item Short-Form Health Survey, version 2.0 (SF-12v2), the Epworth Sleepiness Scale (ESS), and the Snoring, Tiredness, Observed Apnea, High Blood Pressure, Body Mass Index, Age, Neck Circumference, and Gender (STOP-BANG) Questionnaire (SBQ).

**Results:**

Participants with LLL exhibited significantly higher SBQ ( *p* = 0.022) and ESS ( *p* = 0.044) scores compared to those with ULL, indicating an elevated risk of OSA and daytime sleepiness. Furthermore, LLL patients had significantly lower physical ( *p* = 0.020) and mental ( *p* = 0.009) component scores on the SF-12v2, reflecting poorer health-related quality of life. Although PSQI scores were higher in the LLL group, the difference was not statistically significant. Regression analysis showed a significant association between EV and PSQI scores in the LLL group (adjusted *p* = 0.029), highlighting the impact of lymphedema severity on sleep quality.

**Conclusions:**

In this study, LLL was associated with a higher risk of OSA and greater sleep disturbances compared to ULL and controls.

## Introduction

Lymphedema is a chronic and progressive condition characterized by the abnormal accumulation of protein-rich fluid in the interstitial tissues due to impaired lymphatic function. It can be classified as primary, which results from genetic abnormalities within the lymphatic system, or secondary lymphedema, caused by acquired factors such as surgery, trauma, or infections. 1 While this condition is often recognized for its visible swelling and physical discomfort, it is also associated with a wide range of comorbidities that significantly impact patients' overall quality of life (QoL). These comorbidities include chronic pain, reduced mobility, recurrent infections, and psychological distress (i.e. anxiety and depression). 2

An important but often overlooked consequence of lymphedema is its negative impact on sleep quality. 3 Patients frequently report poor sleep due to symptoms like limb heaviness, discomfort, and swelling, which can worsen during nighttime. Limited mobility, fatigue, and the psychological burden of lymphedema further complicate these disturbances. 4 Sleep quality is closely linked to both physical and mental health outcomes, and poor rest can exacerbate other lymphedema-related symptoms, creating a vicious cycle of declining health and reduced QoL. 5 Despite this, few studies have explored the relationship between lymphedema and sleep-related disorders, particularly obstructive sleep apnea (OSA).

As a common sleep disorder, OSA is characterized by repeated upper airway collapse during sleep, leading to intermittent hypoxia, sleep fragmentation, and excessive daytime sleepiness. 6 Growing evidence suggests a strong link between fluid retention and OSA, with nocturnal rostral fluid shift, which is the movement of fluid from the lower limbs to the neck while lying down, playing a central role in its pathogenesis. 7

Previously, we studied the risk of OSA and sleep quality among breast cancer survivors with and without lymphedema. 5 Despite some early reports on the significant prevalence of OSA among individuals with lymphedema, our previous study did not show any greater risk among affected breast cancer survivors. One question raised by our previous research was whether the risk of OSA differs between lower and upper limb involvement.

Individuals with lower-limb lymphedema (LLL) may be particularly susceptible to this phenomenon due to the greater fluid burden in the lower extremities. The redistribution of this fluid to the neck during sleep can lead to upper airway narrowing and increased collapsibility, predisposing LLL patients to OSA. 8 In contrast, patients with upper-limb lymphedema (ULL) may experience a lower risk because fluid accumulation in the upper limbs is less influenced by gravitational fluid shifts during recumbency. However, few studies have directly compared the risk of OSA between LLL and ULL patients, leaving an important gap in understanding how limb involvement influences sleep outcomes in lymphedema populations.

This study aimed to determine whether individuals with LLL face a higher risk of OSA compared to those with ULL and a control group. Additionally, we explored the associated effects on sleep quality, daytime sleepiness, and QoL to provide more understanding of the burden of lymphedema on sleep health.

## Materials and Methods

### Study Design

This cross-sectional study was performed at Motamed Cancer Institute (MCI) in Tehran, Iran. Written informed consent was obtained from all participants. The present study received approval from the MCI following the principles in the Declaration of Helsinki (study Id: 4021721). This study was also reviewed and approved by the Ethical Committee at Shahed University (IR.SHAHED.REC.1403.102).

### Participants and Selection Criteria

This research had three groups, including LLL, ULL, and a control group, aged 30 to 75 years. Both lymphedema patients with and without a previous history of complete decongestive therapy (CDT) were considered eligible. This therapy is performed as the standard treatment in the lymphedema clinic, and consists of manual lymphatic drainage, bandaging, compression, exercise, and skin care.

Patients with active diseases such as cancers, neuromuscular, and psychiatric disorders were excluded. Also, lymphedema patients with other anatomical areas, rather than limbs, were excluded, as well as those with both ULL and LLL simultaneously. Body mass index (BMI) was calculated as a standard part of clinical evaluation. Also, waist and neck circumference were assessed based on previous studies. 5

Lymphedema was diagnosed when the excess volume (EV) of the affected limb exceeded 10% compared to the contralateral limb, or in bilateral cases, exceeded normative thresholds. The following methods were employed to determine the EV and EV percentage (EVP), as previously described. 5 Circumference measurements were taken at predefined intervals along the length of the limb using a nonstretchable measuring tape. For the upper limb, measurements were recorded at intervals of 4 cm starting from the wrist to the axilla. For the lower limb, measurements were taken every 4 cm from the ankle to the groin. Key anatomical landmarks, such as the malleolus and patella (for lower limbs) or the styloid process and olecranon (for upper limbs), were used to ensure consistent positioning during repeated measurements. The limb volume was estimated based on the cylindric formula. 9

The EV was calculated as the difference between the volume of the affected limb and the volume of the unaffected or contralateral limb. Furthermore, EVP was determined to standardize the extent of lymphedema relative to the unaffected limb, with values greater than 10% being considered indicative of clinically significant lymphedema.

For patients with bilateral involvement, limb volumes were compared to normative data adjusted for sex, height, and body weight. Both EV and EVP were calculated using the reference limb volume instead of the unaffected limb. Lymphedema staging was performed based on International Society of Lymphology guidelines and as previously described. 5 9

### Sleep Quality and Risk of OSA Assessment

During the visit, patients provided their demographic information and completed four questionnaires. The first was the Pittsburgh Sleep Quality Index (PSQI), a self-administered instrument for assessing sleep quality and potential disturbance for the last month. It comprises 19 items grouped into 7 components: subjective sleep quality, sleep latency, sleep duration, sleep efficiency, sleep disturbances, use of sleep medications, and daytime dysfunction. Each component is scored from 0 to 3, with the total global score ranging from 0 to 21. Higher scores indicate poorer sleep quality, with a global score ≥6 suggesting significant sleep disturbance. 10 The Persian version of the PSQI has been validated, showing excellent internal consistency (Cronbach's alpha = 0.77) and strong reliability across all components. Its validity in assessing sleep disturbances among Persian-speaking populations has been confirmed in multiple studies. 11

The second tool is known as the 12-Item Short-Form Health Survey, version 2.0 (SF-12v2), which measures QoL concerning mental and physical function. The SF-12v2 assesses two major summary components: Physical Component Summary (PCS), which reflects physical functioning, role limitations due to physical health, pain, and general health perceptions; and Mental Component Summary (MCS), which reflects emotional well-being, role limitations due to emotional problems, social functioning, and vitality. Each component is scored on a scale from 0 to 100, with higher scores indicating better QoL. The SF-12v2 is a shorter alternative to the 36-Item Short-Form Health Survey (SF-36), retaining robust psychometric properties. 12 13 The Persian translation of the SF-12v2 has demonstrated good reliability (Cronbach's alpha = 0.87) and validity in capturing QoL in Persian-speaking populations, including those with chronic conditions like lymphedema. 14

Additionally, the third questionnaire was the Epworth Sleepiness Scale (ESS), which measures daytime sleepiness. The ESS is an 8-item questionnaire that measures daytime sleepiness by evaluating the likelihood of dozing off in various situations, such as sitting quietly, watching television, or during conversations. Each item is scored on a scale of 0 to 3, with a total score ranging from 0 to 24. A score > 10 indicates excessive daytime sleepiness. 15 The Persian version of the ESS has shown excellent reliability (Cronbach's alpha = 0.81) and good construct validity in assessing daytime sleepiness among Persian-speaking individuals. 16

The Snoring, Tiredness, Observed Apnea, High Blood Pressure, Body Mass Index, Age, Neck Circumference, and Gender (STOP-BANG) questionnaire (SBQ) is a simple screening tool for assessing the risk of OSA. It consists of eight yes/no questions based on clinical features and self-reported symptoms: snoring, tiredness during the daytime, observed apnea, and high blood Pressure (STOP), as well as BMI > 35 kg/m ^2^ , age > 50 years, neck circumference > 40 cm, and male gender (BANG). A total score ≥ 3 is considered high risk for OSA. 17 The Persian SBQ has been validated in clinical settings, demonstrating strong sensitivity and moderate specificity for detecting OSA. Its reliability (Cronbach's alpha = 0.77) is adequate for use in screening Persian-speaking populations. 18

### Statistical Analysis

The IBM SPSS Statistics for Windows (IBM Corp.), version 23, was used for statistical analyses in this study. Descriptive analysis was performed for all variables across three groups.

For categorical data, the Chi-squared test was used for the analyses, while Fisher's exact test was applied to variables where more than 20% of expected frequencies were below 5. Continuous variables, such as age, BMI, neck and waist circumferences, and questionnaire scores (PSQI, SF-12v2, ESS, SBQ), were analyzed using either the independent-sample *t* -test or Mann-Whitney's U-test, depending on the distribution of the data.

Statistical analyses were performed using one-way analysis of variance (ANOVA) and Tukey's post-hoc tests for most variables. However, some variables were analyzed using the Welch and Games-Howell post-hoc tests due to significant results from Levene's test, which indicated unequal variances. The *p* -value considered for statistical significance was below 0.05.

## Results

A total of 91 (87 female and 4 male) participants were included in this study, divided into three groups: 32 patients with ULL, 29 with LLL, and 30 healthy controls. All patients with ULL had a previous history of breast cancer, and were considered BCRL. However, the cause of patients with LLL was different. While the majority of LLL patients were due to secondary causes, seven were primary cases.

All lymphedema patients included were unilateral except for two bilateral cases in the LLL group, which both were primary lymphedema. However, three cases in both the ULL and LLL groups were excluded since breast or genital areas were also affected. One lymphedema patient was also excluded, since there was involvement in both upper and lower limbs. More details on the etiologies of LLL patients are provided in Figure 1 .

**Fig. 1:**
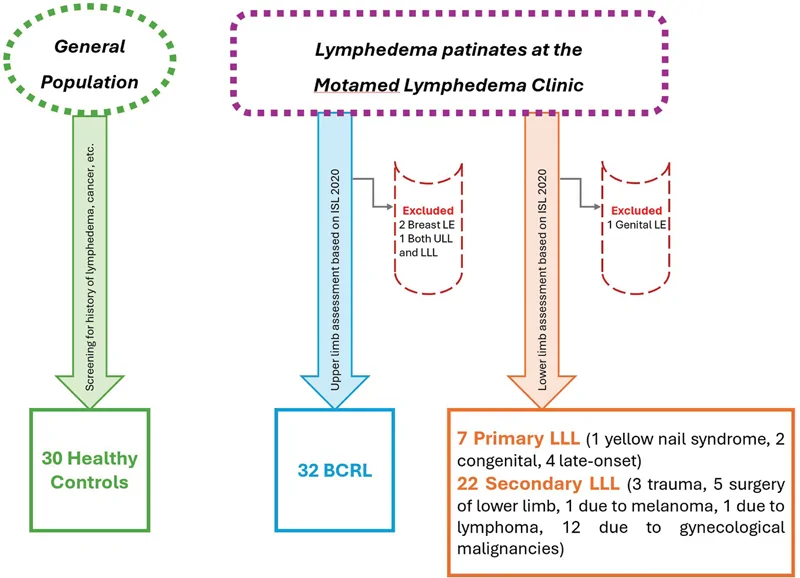
Patient selection process and detailed etiologies of lymphedema.

There was no significant difference between three groups regarding the past medical history including cardiovascular diseases and diabetes. Additionally, while the majority of included ULL and LLL patients had stage 2 lymphedema, there was no difference in the distribution of lymphedema stage among the groups ( Supplementary Table S1 ).

Both EV and EVP were measured in the lymphedema groups. For the ULL group, the mean EV was 427.64 ± 338.79 mL, with an EVP of 13.10 ± 10.86%. For the LLL group, the mean EV was 3,153.28 ± 3,671.03 mL, with an EVP of 10.40 ± 12.23%. The demographic characteristics and questionnaire scores among ULL, LLL, and control groups are summarized in Table 1 . There were no significant differences in age ( *p* = 0.175) or BMI ( *p* = 0.467) across the groups.

**Table 1:** Comparison of the demographic features and questionnaire scores of the study groups

Variables	Control (N = 30)	ULL (N = 32)	LLL (N = 29)	*p* -value			
				Post-Hoc analysis			ANOVA ( *F* )
				Control to ULL	Control to LLL	ULL to LLL	
**Age**	51.90 ± 10.07 (34–68)	47.91 ± 6.85 (32–58)	52.24 ± 13.94 (17–75)	0.175	0.994	0.294	0.116*
**BMI**	26.90 ± 2.76 (21.67–32.89)	28.11 ± 5.02 (19.57–40.81)	30.33 ± 1.71 (21.22–68.78)	0.467	0.149	0.489	0.117*
**PCS**	43.34 ± 10.50 (21.21–63.16)	41.05 ± 8.23 (20.39–56)	35.87 ± 12.46 (7.91–54.35)	0.655	**0.020**	0.137	**0.023** **(3.936)**
**MCS**	51.58 ± 51.58 (34.04–67.73)	45.79 ± 45.79 (18.33–66.62)	42.83 ± 42.83 (13.61–64.16)	0.105	**0.009**	0.551	**0.011** **(4.8)**
**Neck circumference**	36.26 ± 2.57 (32–43)	34.93 ± 3.12 (27–46)	36.69 ± 4.75 (30–48)	0.167	0.906	0.223	0.122*
**Waist circumference**	89.13 ± 13.64 (45–108)	95.40 ± 11.01 (74–125)	96.24 ± 16.35 (67–145)	0.177	0.122	0.970	0.097
**PSQI score**	6.20 ± 2.48 (0–11)	6.38 ± 1.80 (2–10)	7.28 ± 3.32 (3–17)	0.947	0.346	0.406	0.354*
**SBQ score**	1.43 ± 0.97 (0–3)	1.56 ± 1.39 (0–6)	2.34 ± 1.47 (0–5)	0.919	**0.022**	0.054	**0.017** **(4.275)**
**ESS**	4.80 ± 4.34 (0–17)	4.34 ± 3.47 (0–13)	6.97 ± 4.71 (0–16)	0.904	0.122	**0.044**	**0.040** **(3.336)**

**Abbreviations** : BMI, Body Mass Index; ESS, Epworth Sleepiness Scale; LLL, lower-limb lymphedema; MCS, mental component summary; PCS, physical component summary; PSQI, Pittsburgh Sleep Quality Index; SBQ, STOP-BANG Questionnaire; ULL, upper-limb lymphedema.

**Notes:** * Welch test. The data were presented as mean ± standard deviation (range).

Altogether, 34 patients did not receive CDT, while 27 had a history of it. There was no significant difference in the number of patients with previous CDT between the ULL and LLL groups ( Supplementary Table S2 ). Additional demographic data regarding occupation ( *p* = 0.594) and education ( *p* = 0.611) distributions revealed no statistically significant differences among the groups (data not shown).

The PCS scores differed significantly ( *p* = 0.020), with the LLL group showing lower scores compared to the control group. Similarly, the MCS scores were significantly lower in the LLL group ( *p* = 0.009). Individuals in the LLL group had significantly higher SBQ scores ( *p* = 0.022), indicating an elevated risk for OSA compared to the ULL group. The ESS scores were also elevated in the LLL group compared to the ULL group ( *p* = 0.044). Although not statistically significant, PSQI scores were higher in the LLL group. The analysis of the neck and waist circumference measurements showed no statistically significant differences between groups. Moreover, there was no significant difference in PSQI total, SBQ, and ESS scores between lymphedema patients who received CDT or not, regardless of the upper or lower limb being affected ( Supplementary Table S2 ).

Table 2 presents the results of the comparison of categorical data between groups. Sleeping drug consumption varied significantly among the groups ( *p* < 0.001), with the LLL and ULL groups reporting slightly higher rates. While there were no significant differences found in sleep efficiency and high PSQI scores ( *p* = 0.153, *p* = 0.766), SBQ high-risk scores (above two) were significantly more common in the LLL group ( *p* = 0.032). Likewise, ESS scores exceeding ten were more frequently observed in the LLL group ( *p* = 0.010). Additionally, there was no difference in PSQI, SBQ, or ESS between different stages of lymphedema ( Supplementary Table S2 ).

**Table 2:** Comparison of questionnaire outcomes and other categorical variables

Variables		Control (N = 30)	ULL (N = 32)	LLL (N = 29)	*p* -value
**Sleeping drugs**	No	25 (83.33%)	25 (78.13%)	23 (79.31%)	**< 0.001**
	Yes	5 (16.67%)	7 (21.88%)	6 (20.69%)	
**Snoring**	No	26 (86.67%)	24 (75%)	25 (86.21%)	0.391
	Yes	4 (13.33%)	8 (25%)	4 (13.79%)	
**Sleepiness**	No	13 (43.33%)	13 (40.63%)	10 (34.48%)	0.776
	Yes	17 (56.67%)	19 (59.38%)	19 (65.52%)	
**Apnea**	No	25 83.33(%)	28 (87.5%)	22 (75.86%)	0.485
	Yes	5 (16.67%)	4 (12.5%)	7 (24.14%)	
**Sleep duration**	> 7 h	11 (36.67%)	11 (34.38%)	6 (20.69%)	0.051*
	6–7h	13 (43.33%)	16 (50%)	9 (31.03%)	
	5–6 h	3 (10%)	5 (15.63%)	11 (37.93%)	
	< 5 h	3 (10%)	0 (0.0%)	3 (10.34%)	
**Sleep efficiency**	> 85%	19 (63.33%)	25 (78.13%)	13 (44.83%)	0.153*
	75–84%	6 (20%)	3 (9.38%)	10 (34.48%)	
	65–74%	2 (6.67%)	3 (9.38%)	3 (10.34%)	
	< 65%	3 (10%)	1 (3.13%)	3 (10.34%)	
**PSQI**	≤ 5	12 (40%)	11 (34.38%)	9 (31.03%)	0.766
	> 5	18 (60%)	21 (65.63%)	20 (68.97%)	
**SBQ**	≤ 2	25 (83.33%)	27 (84.38%)	17 (58.62%)	**0.032**
	> 2	5 (16.67%)	5 (15.63%)	12 (41.38%)	
**ESS**	≤ 10	28 (93.33%)	31 (96.88%)	21 (72.41%)	0.010*
	> 10	2 (6.67%)	1 (3.13%)	8 (27.59%)	

**Abbreviations** : ESS, Epworth Sleepiness Scale; LLL, lower-limb lymphedema; PSQI, Pittsburgh Sleep Quality Index; SBQ, STOP-BANG Questionnaire; ULL, upper-limb lymphedema.

**Notes:** *Fisher's Exact Test. The data were presented as number (percentage).

Regression analysis results, as outlined in Table 3 , examined the relationships between EV and EVP with questionnaire scores (SBQ, PSQI, and ESS) across ULL, LLL, and combined groups (ULL + LLL). In the combined group, EV showed no significant association with SBQ, PSQI, or ESS scores, both in the unadjusted ( *p* = 0.172, 0.420, and 0.856, respectively) and the BMI-adjusted regression analyses ( *p* = 0.330, 0.281, and 0.985, respectively). Likewise, there were no significant associations for SBQ, PSQI, or ESS scores with EVP in this group ( *p* > 0.05).

**Table 3:** Regression analysis exploring the relationships between the EV and EVP and questionnaire scores unadjusted and adjusted for BMI

Lymphedema		PSQI		SBQ		ESS	
		Unadjusted	BMI-Adjusted	Unadjusted	BMI-Adjusted	Unadjusted	BMI-Adjusted
**All**	**EV**	<0.001 (0.420)	<0.001 (0.281)	<0.001 (0.172)	<0.001 (0.330)	<0.001 (0.856)	<0.001 (0.985)
	**EVP**	0.031 (0.221)	0.032 (0.207)	−0.017 (0.527)	−0.010 (0.724)	0.008 (0.752)	0.011 (0.672)
**ULL**	**EV**	0.003 ( **0.011** )	0.003 ( **0.010** )	−0.001 (0.532)	−0.001 (0.401)	0.001 (0.282)	0.001 (0.273)
	**EVP**	0.077 ( **0.016** )	0.080 ( **0.013** )	−0.023 (0.454)	−0.026 (0.410)	0.048 (0.112)	0.054 (0.075)
**LLL**	**EV**	<0.001 (0.143)	−0.001 ( **0.029** )	<0.001 (0.237)	<0.001 (0.241)	<0.001 (0.309)	<0.001 (0.163)
	**EVP**	−0.133 (0.053)	−0.132 (0.059)	0.013 (0.812)	0.086 (0.296)	−0.102 (0.093)	−0.103 (0.094)

**Abbreviations** : BMI, Body Mass Index; ESS, Epworth Sleepiness Scale; EV, excess volume; EVP, excess volume percentage; LLL, lower-limb lymphedema; PSQI, Pittsburgh Sleep Quality Index; SBQ, STOP-BANG Questionnaire; ULL, upper-limb lymphedema.

**Note:** The data were presented as coefficient ß ( *p* -value).

In the ULL group, EV and EVP were significantly associated with PSQI scores in both unadjusted ( *p* = 0.011 and 0.016, respectively) and adjusted analyses ( *p* = 0.010 and 0.013, respectively). No significant associations were observed for SBQ or ESS scores in this group.

Similarly, in the LLL group, EV was significantly associated with PSQI scores in the adjusted analysis ( *p* = 0.029) but not in the unadjusted analysis ( *p* = 0.143). In the LLL group, EVP showed no significant associations with SBQ, PSQI, or ESS scores in either unadjusted or adjusted analyses ( *p* > 0.05).

Regression analysis results exploring the relationships between the groups (control, ULL, and LLL) and questionnaire scores (SBQ, PSQI, and ESS), unadjusted and adjusted for BMI, are presented in Table 4 . In the unadjusted analysis comparing ULL to LLL, SBQ scores were significantly lower in the LLL group ( *p* = 0.019), a finding that remained significant after adjusting for BMI ( *p* = 0.044). However, no significant differences in SBQ scores were observed when comparing the control group to the ULL group ( *p* = 0.154) or the control group to LLL ( *p* = 0.210).

**Table 4:** Regression analysis exploring the relationships between the groups and questionnaire scores, unadjusted and adjusted for BMI

Questionnaire	Control to ULL		Control to LLL		ULL to LLL	
	Unadjusted	BMI-Adjusted	Unadjusted	BMI-Adjusted	Unadjusted	BMI-Adjusted
**PSQI**	−0.111 (0.804)	−0.027 (0.953)	−0.411 (0.369)	−0.458 (0.333)	-.281 (0.532)	−0.314 (0.491)
**SBQ**	0.667 (0.154)	0.817 (0.088)	−0.588 (0.210)	−0.287 (0.553)	−1.130 ( **0.019** )	−0.978 ( **0.044** )
**ESS**	0.125 (0.779)	0.173 (0.700)	−0.918 ( **0.049** )	−1.302 (0.033)	−1.080 ( **0.020** )	−1.100 ( **0.019** )

**Abbreviations** : BMI, Body Mass Index; ESS, Epworth Sleepiness Scale; LLL, lower-limb lymphedema; PSQI, Pittsburgh Sleep Quality Index; SBQ, STOP-BANG Questionnaire; ULL, upper-limb lymphedema.

**Note:** The data were presented as coefficient ß (p-value).

For PSQI scores, no significant associations were observed across any comparisons in the unadjusted analyses for controls to ULL, controls to LLL, and ULL to LLL. These findings were consistent in the BMI-adjusted analyses, where no significant differences were detected ( *p* > 0.05). The ESS scores demonstrated significant differences when comparing cases and ULL to LLL. In the case of LLL comparison, ESS scores were significantly lower in the LLL group, with *p* -values of 0.049 in the unadjusted analysis and 0.033 after adjusting for BMI. Similarly, in the ULL to LLL comparison, ESS scores were significantly lower in the LLL group, with *p* -values of 0.020 in the unadjusted analysis and 0.019 in the BMI-adjusted analysis. In contrast, no significant differences in ESS scores were observed between the control and ULL groups ( *p* = 0.779 in unadjusted analysis and *p* = 0.700 after adjustment for BMI).

## Discussion

This study investigated the potential association between LLL, ULL, and the risk of OSA. Our findings revealed that individuals with LLL experience significantly higher risks of OSA, as assessed through the SBQ and ESS questionnaires, compared to both ULL patients and controls. Moreover, individuals in the LLL group demonstrated poorer physical and mental health outcomes, as reflected by their lower SF-12v2 scores, indicating greater physical and psychological challenges.

### Lymphedema, Sleep Quality, and Risk of OSA

The observed elevated risk of OSA among LLL patients aligns with previous studies highlighting the role of rostral fluid shifts in OSA pathophysiology. It has been suggested that nocturnal fluid redistribution from the lower extremities to the upper body, including the neck, contributes to the narrowing of the pharyngeal airway and increased susceptibility to OSA. 7 Roux et al. reported a high prevalence of OSA among all patients with lymphedema (74%), particularly in those with lower limb involvement (ULL: 56%, LLL: 85%). 8 Our findings reinforce this association, as SBQ scores, which evaluate clinical indicators of OSA risk, were significantly higher in the LLL group compared to ULL patients and controls. Additionally, daytime sleepiness, measured through the ESS, was markedly more frequent among LLL patients, further supporting the likelihood of sleep-disordered breathing in this population.

Interestingly, while the SBQ and ESS scores differed significantly, other indicators, such as neck circumference and waist measurements, did not show notable differences across groups. This suggests that the anatomical involvement of the lower limbs contributes to sleep problems and higher OSA risk by facilitating nocturnal rostral fluid shifts from the legs to the neck, resulting in upper airway narrowing and increased pharyngeal collapsibility during sleep.

The analysis of sleep quality, as assessed by the PSQI, revealed higher (though not statistically significant) scores in the LLL group. This trend suggests poorer sleep quality among LLL patients compared to ULL patients and controls, further supporting the relationship between lymphedema severity and sleep dysfunction. Additionally, short sleep duration (<6 hours) was more common in the LLL group, which may contribute to increased daytime sleepiness and impaired daytime function.

Moreover, sleep medication use was significantly higher in both LLL and ULL groups compared to controls, suggesting that sleep disturbances are prevalent across lymphedema patients regardless of limb involvement. Previously reported studies also have demonstrated similar findings regarding diminished sleep quality among individuals with lymphedema. Tamam et al. reported more severe sleep problems among breast cancer survivors with more severe stages of ULL. 19 Moreover, a study by Monteiro et al. reported significantly higher rates of sleep disturbances among LLL cases compared to healthy controls. 20

While there are other reports on the diminished sleep quality among individuals with ULL, limited evidence exists regarding sleep problems among LLL, and even more scarce than that is the gap within the comparison between ULL and LLL. Doersam et al. compared symptoms related to sleep difficulties among three groups of head and neck lymphedema, truncal lymphedema, and healthy controls. Sleeping difficulty was significantly higher among participants with truncal lymphedema. 21 These results indicate the possibility of the significance of anatomical involvement in lymphedema within the context of sleep problems such as OSA.

In addition to limb location, we also explored whether a history of CDT was associated with differences in sleep quality or OSA risk. Our analysis showed that the prevalence of prior CDT was similar between ULL and LLL groups, suggesting that it did not contribute to the observed differences in outcomes. Furthermore, PSQI, SBQ , and ESS scores did not significantly differ between patients with and without CDT. These findings indicate that the higher burden of sleep disturbance and OSA risk among LLL patients is unlikely to be explained by prior treatment status. Nonetheless, we acknowledge that the present study did not evaluate ongoing adherence to compression therapy or other management strategies, which may still influence outcomes and warrant investigation in future research.

Our study also highlights the significant impact of lymphedema on both physical and mental health. The PCS and MCS scores from the SF-12v2 questionnaire were significantly lower among LLL patients compared to controls. This suggests that individuals with LLL face greater physical disability, fatigue, and mental health challenges, likely exacerbated by sleep disturbances and reduced QoL. These findings are consistent with prior literature that underscores the physical and psychosocial burden of lymphedema, including decreased mobility, chronic pain, fatigue, financial toxicity, and social stigma. 22 23 Notably, while ULL patients also reported some sleep disturbances and QoL impairments, the LLL group exhibited more severe outcomes, meaning higher risk of OSA and lower QoL. This discrepancy may be attributable to the greater gravitational impact of fluid accumulation in the lower limbs, which exacerbates functional limitations and fluid redistribution during sleep. As a result, LLL patients may experience more pronounced nocturnal symptoms, such as airway obstruction and disrupted sleep patterns. While emerging evidence indicates that gravity could cause resistance towards pharyngeal expansion and possibly contribute to the pathophysiology of central sleep apnea, 24 the exact mechanism is poorly understood among individuals affected with lymphedema.

### Potential Mechanisms for Higher Risk of OSA

Several pathophysiological mechanisms may explain the increased risk of OSA among LLL patients. First, as previously mentioned, fluid shifts from the lower extremities to the upper body during recumbency play a central role in OSA development. The redistribution of this fluid to the neck during sleep can result in upper airway narrowing, increased collapsibility, and eventual obstruction, particularly during REM sleep when muscle tone is reduced. 25

The most plausible explanation for the higher OSA risk in LLL patients compared to ULL patients is the volume and gravitational impact of fluid accumulation. In LLL, significant fluid builds up in the lower limbs due to impaired lymphatic drainage. During sleep, especially in the supine position, this excess fluid is redistributed upward (rostrally) toward the neck and thoracic regions. This nocturnal fluid shift increases tissue edema in the upper airway, contributing to airway narrowing and increased collapsibility. 8 In contrast, ULL primarily involves the upper limbs, where fluid volume is substantially less, and gravitational shifts during recumbency are less pronounced. As a result, ULL patients experience reduced fluid redistribution to the neck, which likely explains the lower OSA risk observed in this group. 5

The larger volume of edema in LLL patients can exacerbate OSA risk through systemic effects beyond rostral fluid shift. Excessive lower limb fluid accumulation may result in elevated central venous pressure (CVP) and systemic inflammation, both of which are associated with upper airway edema. Elevated CVP promotes fluid leakage into soft tissues, including the neck, worsening pharyngeal collapsibility during sleep. 26 By contrast, ULL typically involves less severe lymphatic dysfunction and lower overall fluid burden, which limits its impact on systemic hemodynamics. This may explain why SBQ and ESS scores in the ULL group were closer to those of the control group than the LLL group.

Second, systemic inflammation, which is often elevated in lymphedema, may contribute to sleep-disordered breathing. Recent findings have revealed the significant role of inflammatory and fibrotic pathways within the pathophysiology of lymphedema. 27 28 This condition is characterized by chronic inflammation, lymphatic dysfunction, and impaired immune regulation, which can exacerbate systemic edema and airway inflammation. Inflammation-induced airway narrowing and reduced neuromuscular control of the pharynx may further predispose individuals with LLL to OSA.

Third, the physical limitations associated with LLL, including decreased mobility and increased sedentary behavior, can contribute to weight gain and obesity, both of which are significant risk factors for OSA. 29 While BMI did not differ significantly among the groups in our study, it is possible that other obesity-related factors, such as fat distribution or visceral fat accumulation, may play a role in sleep disturbances among LLL patients. 30 This condition is associated with significant physical limitations, including reduced mobility, pain, and heaviness in the lower limbs. These factors often lead to a sedentary lifestyle, which in turn predisposes individuals to weight gain, increased visceral fat, and a higher risk of OSA. On the other hand, ULL does not impact ambulation and physical activity to the same extent. As a result, ULL patients may maintain better functional capacity, which reduces their risk of secondary factors such as obesity and deconditioning that exacerbate OSA risk ( Fig. 2 ).

**Fig. 2:**
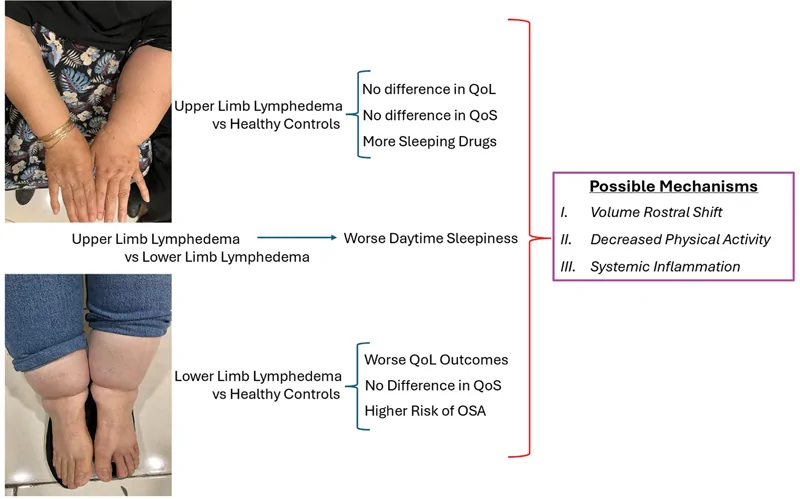
Possible mechanisms of sleep disturbances in lymphedema.

### Strengths and Limitations

This study has several strengths, including its inclusion of both LLL and ULL groups, comprehensive assessment of sleep-related symptoms, and use of validated questionnaires for OSA risk and QoL evaluation. However, some limitations should be acknowledged. First, the cross-sectional design limits causal inferences between lymphedema and OSA risk. Longitudinal studies are needed to assess the temporal relationship between fluid shifts, sleep disturbances, and OSA development in lymphedema patients.

Second, the reliance on subjective questionnaires, rather than objective measures such as polysomnography, may introduce reporting bias. Future studies incorporating polysomnography or home sleep apnea testing could provide more robust diagnostic confirmation.

Third, the sample size may have limited the statistical power to detect significant differences in certain variables, such as PSQI scores. Fourth, another limitation is that although prior CDT history was assessed, we did not evaluate adherence to ongoing compression therapy or other management strategies, which may influence outcomes.

Lastly, it should be noted that the limited number of primary and bilateral lymphedema, as well as those with involvement of the truncal or genital area, made subgroup analyses impossible. Further comparisons among any of these groups would bring more understanding to the current uncertainties of sleep quality and risk of OSA among individuals affected by lymphedema.

## Conclusion

The present study demonstrates that individuals with LLL are at a significantly higher risk of OSA compared to those with ULL and control subjects. This increased risk is accompanied by poorer physical and mental health outcomes, as well as greater sleep disturbances. These findings highlight the need for routine OSA screening and comprehensive management strategies for patients with LLL. Addressing sleep-related issues and improving overall QoL should be prioritized in the clinical care of lymphedema patients.
