# Supplementary material for: Is There a Higher Risk of Obstructive Sleep Apnea among Individuals with Lower-Limb Compared to Those with Upper-Limb Lymphedema?
Source: Sleep Sci. 2026 Nov 3;19(3):1–10. doi: 10.1055/s-0046-1819596 (PMC13730673; doi:10.1055/s-0046-1819596)
Supplement: Supplementary file 1 [file 10-1055-s-0046-1819596-s250427.pdf]

Supplementary File

Table 1 Comparison of the lymphedema stage, CDT history, and, past medical history among study groups

Group		Control (N = 30)	ULL (N = 32)	LLL (N = 29)	Total (N = 91)	*p*-value
**Lymphedema Stage**	**Stage 1**	-	6 (43%)	8 (57%)	14	0.253
	**Stage 2**	-	20 (63%)	12 (37%)	32	
	**Stage 3**	-	6 (40%)	9 (60%)	15	
**History of CDT**	**Yes**	-	16 (47%)	18 (53%)	34	0.343
	**No**	-	16 (59%)	11 (41%)	27	
**Cardiovascular Diseases**	**Yes**	19 (28%)	28 (42%)	20 (30%)	67	0.077
	**No**	11 (46%)	4 (17%)	9 (27%)	24	
**Metabolic Diseases**	**Yes**	19 (35%)	21 (39%)	14 (26%)	54	0.334
	**No**	11 (30%)	11 (30%)	15 (40%)	37	
**Sex**	**Male**	0 (0%)	0 (0%)	23 (100%)	23	< 0.001
	**Female**	30 (44%)	32 (47%)	6 (9%)	68	

Abbreviations: CDT, Complete Decongestive Therapy; **LLL,** Lower Limb Lymphedema; **N,** Number; **ULL,** Upper Limb Lymphedema.

Table 2 Comparison of QOS and risk of OSA in study subgroups based on the lymphedema stage and history of CDT

Group		PSQI	SBQ	ESS
**Lymphedema Stage**	**Stage 1** (N = 14)	6.00 ± 2.39	1.71 ± 1.38	5.71 ± 3.79
	**Stage 2** (N = 32)	6.69 ± 2.81	2.13 ± 1.56	5.28 ± 4.35
	**Stage 3** (N = 15)	7.80 ± 2.40	1.73 ± 1.39	6.13 ± 4.79
	***p*-value**	0.179	0.576	0.816
**History of CDT**	**Yes** (N = 27)	6.59 ± 2.29	2.07 ± 1.32	5.93 ± 3.64
	**No** (N = 34)	6.97 ± 2.93	1.82 ± 1.58	5.32 ± 4.77
	***p*-value**	0.585	0.513	0.590

Abbreviations: ESS, Epworth Sleepiness Scale; **N,** Number; **OSA,** Obstructive Sleep Apnea; **PSQI,** Pittsburgh Sleep Quality Index; **QOS,** Quality of Sleep; **SBQ,** STOP-Bang Questionnaire.

 © 2026. The Author(s).
